# A Complementary Fusion-Based Multimodal Non-Destructive Testing and Evaluation Using Phased-Array Ultrasonic and Pulsed Thermography on a Composite Structure

**DOI:** 10.3390/ma17143435

**Published:** 2024-07-11

**Authors:** Muhammet E. Torbali, Argyrios Zolotas, Nicolas P. Avdelidis, Muflih Alhammad, Clemente Ibarra-Castanedo, Xavier P. Maldague

**Affiliations:** 1 School of Aerospace, Transportation and Manufacturing, Cranfield University, Cranfield MK43 0AL, UK; ebubekir.torbali@cranfield.ac.uk (M.E.T.); a.zolotas@cranfield.ac.uk (A.Z.); np.avdel@cranfield.ac.uk (N.P.A.); alhammadmuflih@gmail.com (M.A.); 2 Department of Electrical and Computer Engineering, Universite Laval, Quebec, QC G1V 0A6, Canada; xavier.maldague@gel.ulaval.ca

**Keywords:** non-destructive testing, fusion in NDT & E, combinations of NDT & E, multimodal NDT& E, phased-array ultrasonic testing, pulsed thermography, composite structures

## Abstract

Combinative methodologies have the potential to address the drawbacks of unimodal non-destructive testing and evaluation (NDT & E) when inspecting multilayer structures. The aim of this study is to investigate the integration of information gathered via phased-array ultrasonic testing (PAUT) and pulsed thermography (PT), addressing the challenges posed by surface-level anomalies in PAUT and the limited deep penetration in PT. A center-of-mass-based registration method was proposed to align shapeless inspection results in consecutive insertions. Subsequently, the aligned inspection images were merged using complementary techniques, including maximum, weighted-averaging, depth-driven combination (DDC), and wavelet decomposition. The results indicated that although individual inspections may have lower mean absolute error (MAE) ratings than fused images, the use of complementary fusion improved defect identification in the total number of detections across numerous layers of the structure. Detection errors are analyzed, and a tendency to overestimate defect sizes is revealed with individual inspection methods. This study concludes that complementary fusion provides a more comprehensive understanding of overall defect detection throughout the thickness, highlighting the importance of leveraging multiple modalities for improved inspection outcomes in structural analysis.

## 1. Introduction

Composites made of carbon and glass fibers (CFRPs and GFRPs) have a high strength-to-weight ratio and are resistant to corrosion; these properties make them useful in many applications. Ultrasonic inspection is one of the most common techniques used to check the quality of these composites in terms of any anomaly, discontinuity, or defect to avoid a disastrous malfunction. Conventional ultrasonic testing, which relies on single-element transducers, is constrained by issues such as significant attenuation and a low signal-to-noise ratio (SNR). The propagation of waves in anisotropic composite structures is complicated, and the presence of random scattering and substantial attenuation of ultrasonic waves significantly diminishes the likelihood of detecting defects [1].

Phased-array ultrasonic testing (PAUT) can effectively address the issues of a low SNR and inadequate resolution by employing the capacity to focus and manipulate the ultrasound beam at specific depths and angles [2]. This approach uses the same theory, which is variations in pulse velocity and transmission loss across materials, but fires several elements simultaneously to produce ultrasound waves. Ultrasonic waves efficiently travel through dense media, but discontinuities result in diffraction or refraction. As a result, this technique allows for identifying and assessing the size and orientation of discontinuities within the material [3]. PAUT has been utilized highly effectively in recent decades to examine multilayer composite structures [4,5,6,7,8,9], as well as to conduct inspections of additively produced parts [10,11], weldings [12,13,14,15], pipes [16,17], rail constructions [18,19,20], and other structures for various purposes [21,22,23].

Pulsed thermography (PT) is a non-invasive and contactless inspection method that can identify sub-surface defects without causing damage to the tested materials. The inception of PT may be traced back to the mid-20th century [24] when scientists conducted research on the application of infrared thermography in industrial settings. Basically, the introduction of short pulses of heat allowed for enhanced detection of sub-surface anomalies by capturing the transient thermal responses of the materials [25]. Pulsed thermography was adopted by the aviation and aerospace sectors to detect hidden flaws, including delaminations, voids, and impact damage, in composite structures, which is essential for lightweight, high-strength applications [26,27].

Pulsed thermography has demonstrated significant utility in the domain of composite materials [28,29,30,31] for overcoming the obstacles introduced by the heterogeneous characteristics of composite structures. The method’s remarkable capacity to identify even the most subtle temperature variations and thermal patterns has made it a vital resource for guaranteeing quality control and evaluating the structural soundness of composite materials.

The aforementioned inspection methods possess particular benefits and drawbacks as a result of their varying abilities and inherent attributes. For instance, ultrasonic inspection encounters challenges when it comes to detecting surface defects [32], whereas thermography can not identify deep-placed abnormalities as a result of the anisotropic nature of composites, which greatly affects heat diffusion (with larger depths being more affected) [33,34]. The concept of integrating these two distinct approaches to enhance defect traceability in composite structures has not yet been thoroughly explored. It has the potential to deliver improved resolution for determining the size and location of a defect, but it also raises specific challenges. An essential aspect to consider is the registration or alignment of inspection data/images, which poses a significant challenge. However, there is a scarcity of research in the literature that compares the registration of data/images in non-destructive testing with the concept of fusing these two NDT & E techniques.

The application of multimodal image registration (MMIR) has already become essential in several research domains and complex activities, such as object detection or medical image fusion [35,36]. The primary objective of this approach is to effectively identify and collect a diverse set of physical attributes from several modalities. This process ultimately leads to the creation of more comprehensive scene representations through the utilization of image registration and fusion techniques. The use of advanced recognition, tracking, and detection techniques enables the identification of contrasts or correlations within the source images while also facilitating the incorporation of additional information across several modalities to generate three-dimensional reconstruction [37].

In the historical development of image registration studies, two predominant strategies are commonly employed: area-based and feature-based methods. Area-based techniques utilize metrics of similarity as a means to direct the alignment and optimization of images, whereas feature-based methods concentrate on the extraction of distinguishing features, their matching, and the subsequent execution of changes for the purpose of image registration. Feature-based methods are commonly used due to their versatility and robustness across a wide range of applications. In recent years, significant advancements in deep learning have brought about a transformative impact on image matching. These advancements have introduced data-driven approaches as viable alternatives to conventional methods for tasks such as information extraction, similarity evaluation, and the estimation of transformation parameters. However, the pursuit of a generally applicable image registration pathway that offers enhanced precision, resilience, and efficiency continues to be an enduring obstacle.

The registration framework is commonly regarded as an optimization issue, with the objective of boosting similarity or minimizing cost. It might be useful to express it as follows, with Equation (1) [38]:

(1) $$T_{g}\left(.\right)=arg\max_{T_{g}\left(.\right)}\rho \left(I_{r},T_{g}\left(I_{t}\right)\right)$$

where $T_{g}\left(.\right)$ is a parametric transformation carried out on an input image (target), $I_{t}$, to optimize the similarity measurements with a reference image, $I_{r}$. In this way, the registration might be defined as a process of transformation for the input images with respect to a reference, aligning the best-matching features. Such a process might be considered a precondition for a fusion application in any field.

In the field of medical image processing, where registration finds widespread use with computer vision [39], it might be considered of paramount significance to provide precise alignment to distinguish between classes (i.e., healthy or not). However, this is still not exploited in the NDT & E imaging field because these systems are commonly utilized individually for defect detection problems. Considering the combination or confirmation of these NDT & E systems, the amount of registration approaches applied is only limited to several calibration initiatives [40,41,42,43] for X-ray scanning applications. As an example, the article in [40] proposes a practical geometry calibration method using a volume-to-image registration scheme for industrial X-ray tomography systems without a reference item. The authors calculated (dis)similarity measurements, such as the sum of squared differences (SSD) or pattern intensity (PI), to use in optimization algorithms for calibration. Their study claims that the precise calculation of geometrical characteristics might be achieved by utilizing projection data and the numerical model of the object. Their experimental findings indicate that this method produces precise outcomes, even when the initial conditions deviate significantly from actuality.

An object-based geometric alignment strategy has also been introduced in [41] for X-ray scanning. These authors used a phantom, which has fourteen little point-like objects, with four on each of three orthogonal lines and two on a diagonal line. This test item aims to offer distinct answers for various scanner geometries, even when some measurement data are lost due to overlapping locations in the calibration scan. Sun et al. [42] have proposed a four-point phantom using only one projection, which prevents the introduction of mistakes during rotation. Moreover, the authors of [43] offered a similar point-like object using the Fourier transform on the projection-orbit data. Reconstructed micro-CT images show how well the related misalignment correction works at getting rid of image artefacts.

This study has employed a center-of-mass-based registration strategy to minimize alignment uncertainty in order to enhance fusion applications and prevent misclassification in regions of health or damage. This is similar to the medical field’s efforts to save healthy tissues [44]. However, it is not possible to have images from the same scenes in case of different NDT & E inspections, contrary to medical imaging. NDT & E images have different scenes, scales, and distances depending on the inspection modalities. Because of this difficulty, it is challenging to successfully capture and match similar features from different sources of imaging modalities. Therefore, using the center-of-mass aspect, a total transformation matrix has been obtained from the alignment of individual defects. Here, the full-area capture approach in experiments facilitated the calculations for transformation, providing almost aligned images from whole surfaces of specimens. In addition, the maximum, averaging, DDC, and wavelet decomposition combination rules were utilized in this study to obtain complimentary information from PT and PAUT through the application of fusion. The combination of these two specific methodologies and the implementation of the registration application for this purpose might be regarded as novel contributions to the literature. The Section 2 of this study will provide a description of the specimen and the defect. Furthermore, it will provide thorough information on the procedures used for each phase in the experiments, registration, processing, and fusion. The Section 3 will include the findings of each listed phase, along with corresponding criticisms. The Section 4 will discuss the conclusion and directions for future works.

## 2. Methodology

### 2.1. Material Description and Inspection Details

**Figure 1:**
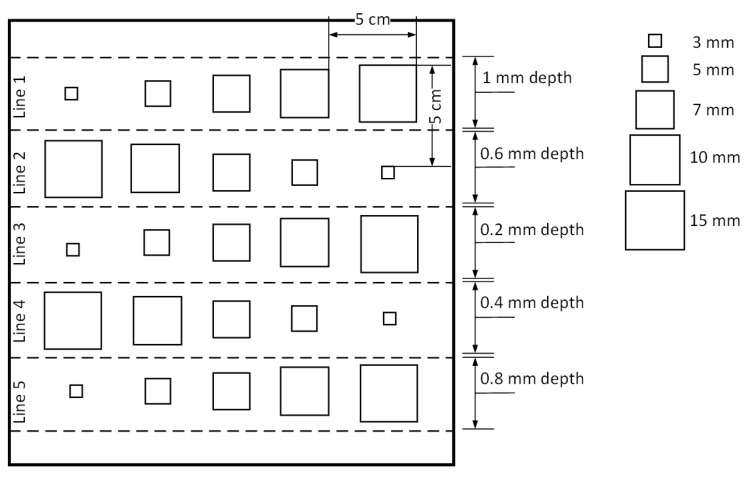
Specimen specifications.

This study aims to focus on artificially produced delamination defects for the assessment of composite structures. The delamination specimen is a CFRP composite structure with 2 mm thickness and 10 plies. It includes five different sizes of Teflon square inclusions (3, 5, 7, 10, and 15 mm) in various depths, as shown in Figure 1. The specimen has a total size of 300 × 300 mm. For the purpose of facilitating analysis during inspection and comparison, it has been assumed that the lines are divided into five separate groups based on their thickness. This implies that the initial line will be designated as ‘Line 1’, and subsequent lines will follow this numbering pattern. The inspection results are allocated according to this numbering pattern and given in Section 3. Table 1 summarizes the specific features of the delamination specimen [45].

### 2.2. Experimental Details of PAUT

**Figure 2:**
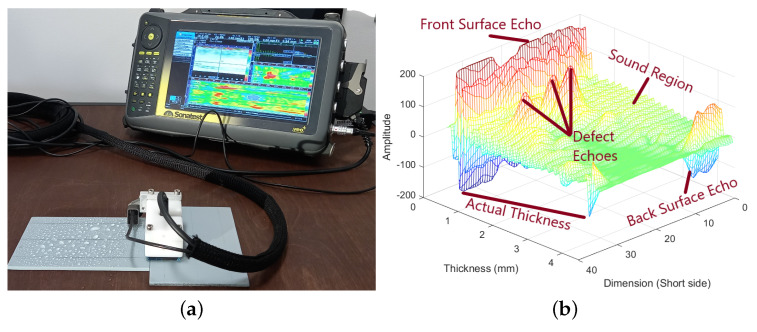
NDT & E experimental setup and representative demonstration of base signals for PAUT. (**a**) PAUT transducer and equipment setup for inspection; (**b**) a symbolic PAUT frame of signals including defective peaks and sound region through the thickness.

The PAUT data acquisition was performed using a Sonatest PA X3 series probe (Sonatest, Milton Keynes, UK), which is a 10 MHz frequency and 64-element linear phased-array transducer with a pitch of 0.6 mm. The probe is equipped with a custom-developed flat elastomer wedge (Figure 2a). The process of ultrasonic scanning involved the use of a collection of eight active elements, referred to as the active aperture. The equation to determine the total number of A-scans that may be obtained with a single line scan of a phased-array (PA) transducer is given by M=(numberofelements−activesubaperture+1). Hence, in the case of a 64-element transducer with 8 elements selected as an active sub-aperture, the total count of A-scans may be determined as M=64−8+1=57. This signifies a line scan of 34.2 mm in each 1 mm frame (through the scanning distance), similar to the shorter side of an image. The study conducted by Mohammadkhani [6] served as a basis for localizing the front, back, and defect echoes and implementing thresholding to extract defects.

The PAUT experiments were conducted on a dry, flat surface in order to prevent any potential interference due to excessive water presence beneath the specimens. The probe was manipulated across the surface of the specimen, using sprayed water as a means of coupling, while an encoder tracked the probe to calculate the distance of the scanning process. The specimens were subjected to guidelines based on the transducer acquisition size to guide a straight scan. Full surface coverage has also been adopted here to simplify the process of alignment and further integration. A representative frame of A-scan signals from a defective point is presented in Figure 2b to demonstrate front and back surface echoes as well as to show the thickness of the specimen.

### 2.3. Experimental Details of PT

In the pulsed thermography experiments, a 1280 × 1028 pixel array FPA infrared camera (FLIR 8501sc (FLIR Systems, Inc., Wilsonville, OR, USA), 3–5 μm) was used to record all of the thermogram sequences of delamination [45]. Two photographic flashes (6.4 kJ each; Balcar FX 60 (London, UK)) triggered by a 5 ms pulse were used in these tests. The specimens were arranged with the camera at a sufficient distance in order to cover the whole surface and the flashes were about 10 cm away, creating a 45° angle with regard to the specimen surface; the exact distance varied depending on the test method (transmission or reflection).

**Figure 3:**
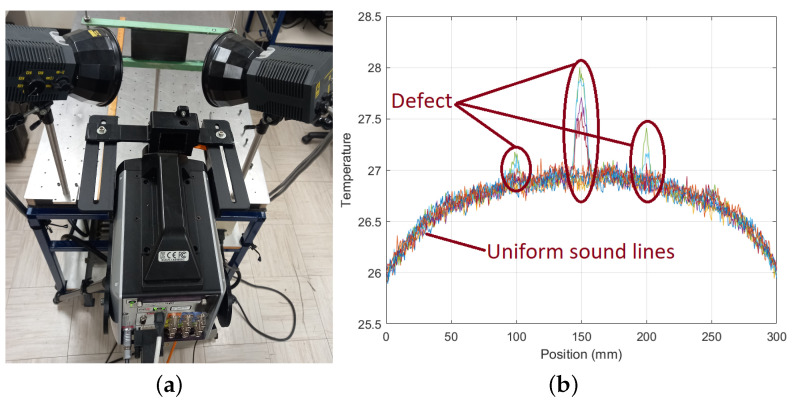
NDT & E setup for PT and a representative demonstration of PT lines. (**a**) PT setup in reflection mode; (**b**) arbitrary line profiles showing heat differences on defective and sound parts.

Briefly, all PT experiments were implemented in reflection mode (the camera was positioned parallel to the heating source, as seen in Figure 3a). The thermography data included an image sequence of over one thousand shots captured throughout the cooling phase. To streamline the registration procedure, the acquisition setup was designed to capture imaging from the whole surface. One of the primary challenges in accurately depicting issues lies in identifying the image that best exemplifies the defect. To succeed in this, adaptive thresholding, which adjusts the threshold level based on a histogram analysis of the image pixels, was employed. Essentially, a histogram represents the frequency distribution of pixel values, plotting the occurrence of each intensity level against its corresponding count or frequency. This procedure makes it feasible to accurately assess the magnitude of any detected imperfections observed in line profiles. An example line profile demonstration to simplify understanding of the discrimination of defective and sound areas is given in Figure 3b. The shape of the profiles might be attributed to the two heat sources on both sides, meaning slightly higher temperatures in the middle.

### 2.4. Image Binarization

PAUT and PT defect detection was performed using the aforementioned techniques above, and the images, which include defect marks with a sound background, were obtained. Then, it was required to process the images in terms of getting similar forms for individual sources. Binary images were generated to discriminate defective and sound regions in the images. Image binarization is a fundamental approach in image processing that converts grayscale or color pictures into binary images. This procedure classifies pixels as either foreground or background, depending on their intensity levels, applying global, local, or dynamic thresholding [46]. This method is essential for various computer vision applications, such as object identification, segmentation, and feature extraction. Binarization eases image processing by decreasing complexities and directing attention towards areas of importance.

The Canny edge detection algorithm is a well-known approach used in computer vision and image processing to help achieve image binarization. Canny edge detection is a method used to identify edges in an image by detecting noticeable variations in intensity, which often represent the borders of objects or prominent features. This approach comprises numerous essential phases, such as Gaussian smoothing to minimize noise, gradient computation to determine edge intensity and orientation, non-maximum suppression to refine identified edges, and edge tracking via hysteresis thresholding [47,48]. The following equations refer to the basics of these phases [46]. Equation (2) indicates a basic Gaussian smoothing operation. For the smoothed (x,y) position, the gradient can be represented by Equation (3) as magnitude and Equation (4) as orientation.

(2) $$S\left(x,y\right)=\frac{1}{2\pi \sigma^{2}}e^{-\frac{x^{2}+y^{2}}{2\sigma^{2}}}$$

(3) $$mag\left(▿f\right)=\sqrt{G_{x}^{2}+G_{y}^{2}}$$

(4) $$\theta \left(x,y\right)=\arctan\left(\frac{G_{y}}{G_{x}}\right)$$

**Figure 4:**
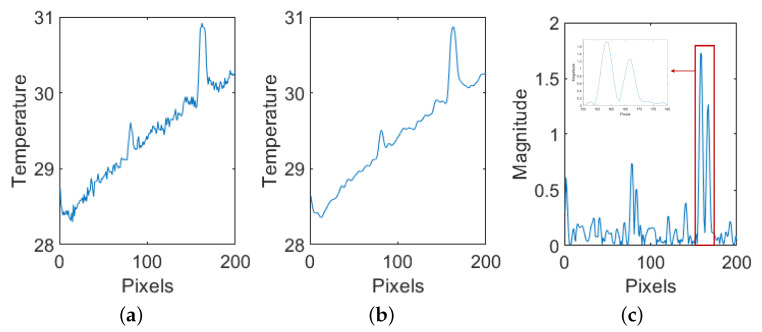
Canny edge detection performance criteria demonstrations of an example defected PT line profile. (**a**) Representative noisy line from a PT image; (**b**) stronger responses at edge positions in the smoothed line; (**c**) good localization with local maximums in a reasonable neighborhood.

The Canny edge detection technique produces a binary image that exclusively displays the identified edges, therefore offering a reduced depiction of the underlying structure of the original image. Figure 4 represents the basic steps of Canny edge detection for one of the defected lines in PT, showing performance criteria. It exhibits good detection as seen by the stronger peaks after Gaussian smoothing (Figure 4b), confirming the first criterion. Moreover, the localization is confirmed as it identifies maxima at the defective pixels and ensures that each maximum is unique within a suitable proximity (Figure 4c) [49].

### 2.5. Details of Registration Procedure

Registration is an essential step in order to align our multimodal application and gain a more comprehensive insight into subsequent activities. It is important to note that our inspections have been conducted utilizing the whole-surface coverage aspect in order to facilitate the registration process with straightforward translations. Then, it has been supported by basic geometric transformation operations based on the mass centers of defected areas. Geometric transformations alter the spatial configuration of pixels inside an image. The process of geometric transformation in digital images involves two fundamental operations: altering the spatial coordinates and assigning intensity values to the altered pixels through intensity interpolation. The whole-coverage approach, which is used in this study, allows only the application of spatial configuration changes.

For a given (*x*,*y*) coordinate, the corresponding coordinate (*x*′,*y*′) for the affine transformation of a 2D image can be expressed as

(5) $$\left[\begin{array}{c} x^{\prime }\\ y^{\prime }\\ 1 \end{array}\right]=A\left[\begin{array}{c} x\\ y\\ 1 \end{array}\right]=\left[\begin{array}{ccc} a_{11} & a_{12} & a_{13}\\ a_{21} & a_{22} & a_{23}\\ 0 & 0 & 1 \end{array}\right]\left[\begin{array}{c} x\\ y\\ 1 \end{array}\right]$$

An affine transformation in 2D is distinguished by its ability to maintain the integrity of points, straight lines, and planes. The image can undergo various transformations, such as scaling, rotating, translating, or shearing, based on the specific values assigned to the members of matrix A. Table 2 expresses the matrix A according to its basic equations and schematic projection [50].

In detail, the center of mass of each defect in a certain line was first determined. These points were then matched with the corresponding points in the other inspection result. Then, the transformation matrix (A) was derived to align the obtained points. Finally, the transformation was applied to the PT images, and the PAUT image was used as a reference. These steps were repeated for each line, and the registration was completed.

### 2.6. Fusion Procedure

The individual inspection systems provided us with detection results thanks to their imaging abilities. This provided information was then processed to differentiate sound and defective areas, implementing basic and simple methods. Here, the primary objective was to boost the overall detection performance using fusion approaches, as opposed to alternative approaches that may enhance the detection rate of individual sources. This means that this study aims to investigate how a fusion can offer complementary insight into multimodal inspection systems while dealing with a specific set of delamination defects.

The goal of complementary applications is to provide a deeper understanding of the underlying phenomenon by capitalizing on the strengths that each information source brings. The maximum fusion rule is crucial for complementary applications because it enables the integration of diverse information from multiple sources while maintaining the highest level of confidence in the resulting fused data. For our case, let us consider two images represented as matrices *A* and *B*, where each element $A_{ij}$ and $B_{ij}$ corresponds to the intensity value at pixel coordinates (i,j) in the respective images. The fused image *F* using the maximum fusion rule is computed by taking the element-wise maximum of the corresponding pixel values from sources, represented as follows:

(6) $$F_{ij}=\max\left(A_{ij},B_{ij}\right)$$

Weighted averaging is another technique employed to integrate data from various sources, wherein the weighting of each source’s contribution is determined by its trustworthiness or significance. In other words, the fused output is equal to the weighted sum of the individual measurements or observations provided by the sources, divided by the sum of the weights. This rule allows sources with higher weights to have a greater influence on the fused output. Similarly, it might be represented as fused image, *F*:

(7) $$F_{ij}=w_{A}\;\cdot \;A_{ij}+w_{B}\;\cdot \;B_{ij}$$

where the weights $w_{A}$ and $w_{B}$ determine the contribution of each image to the fused result.

In this work, depth-driven combination (DDC) is proposed as a combination of rules to compare with the maximum fusion rule and weighted averaging methods. The depth of particular defects, such as delamination in our example, is a crucial attribute that can vary in detectability according to the inspection methods used. To clarify, PT is a type of inspection that is effective on the surface or in close proximity to it. In contrast, phased-array ultrasonic inspection technology possesses the capability to detect defects in deep layers based on the frequency employed. The DDC method identifies two critical depth values, resulting in three intervals for the combination of three fusion rules. Considering *A* is the PA ultrasonic inspection image, which acts as the depth information provider for our case, the fused image, *F*, is as follows:

(8) $$F_{ij}=\left\{\begin{array}{cc} B_{ij}, & \mathrm{if}\;\mathrm{depth}(A_{ij})<0.3\\ w_{A}\;\cdot \;A_{ij}+w_{B}\;\cdot \;B_{ij}, & \mathrm{if}\;0.3\leq \mathrm{depth}(A_{ij})<0.7\\ A_{ij}, & \mathrm{if}\;\mathrm{depth}(A_{ij})\geq 0.7 \end{array}\right.$$

The critical depth values of 0.3 mm and 0.7 mm were selected based on the visual examination of the source detection images. It was obvious that the detection of different sources exhibited distinct characteristics both above and below these limits.

Lastly, wavelet decomposition is a technique used to decompose an image into different frequency bands, allowing for both localization in space and frequency. Fusion at each level of decomposition involves combining corresponding coefficients from the decomposed images. The fused image, *F*, is represented by

(9) $$F_{ij}=\sum_{k=1}^{N}\left(\alpha_{k}\;\cdot \;A_{ij}^{\left(k\right)}+\left(1-\alpha_{k}\right)\;\cdot \;B_{ij}^{\left(k\right)}\right)$$

Here, *N* represents the number of decomposition levels, which was chosen as five in this study’s experiments. $A_{ij}^{\left(k\right)}$ and $B_{ij}^{\left(k\right)}$ represent the wavelet coefficients at level *k* of the source images, respectively. Finally, $\alpha_{k}$ represents the fusion coefficients for level *k*, determining the contribution of the source images at each decomposition level.

## 3. Results and Discussion

### 3.1. Image Registration

**Figure 5:**
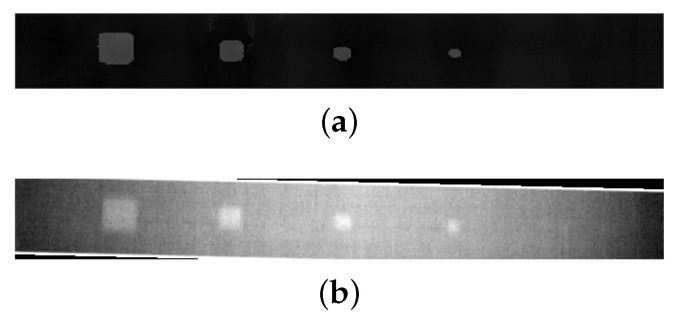
Representative registered image pair based on center-of-mass matching. (**a**) Fixed PAUT image as a reference for required transformation. (**b**) Registered PT image, which has a slight rotation.

The experimental data collection was conducted meticulously to ensure complete coverage of the specimens, as previously stated. The data/images provided were nearly aligned, with just a minor transformation required. The registration process was successfully completed by applying affine transformation using the mass centers of the observed inclusions. Following this stage, we will assume that all data/images from individual sources have been precisely aligned for subsequent procedures. Figure 5 gives this registration in a visual format, illustrating the transformation arising from individual sources. Prior to registration, the PAUT image was selected as a reference image to minimize the computational time required for subsequent processes. The reason for this is that the PAUT image contains depth information, making it advisable to establish it as a permanent reference for future utilization of its depth value.

### 3.2. Defect Detection

**Figure 6:**
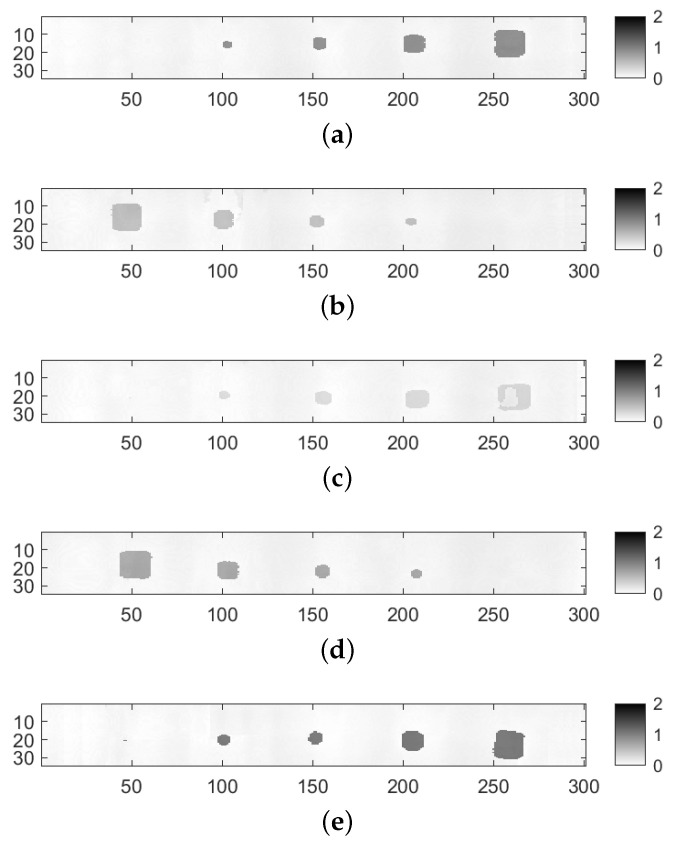
PAUT inspection results for delamination lines with different thicknesses. (**a**) PAUT inspection result for Line 1: 1 mm thickness; (**b**) PAUT inspection result for Line 2: 0.6 mm thickness; (**c**) PAUT inspection result for Line 3: 0.2 mm thickness; (**d**) PAUT inspection result for Line 4: 0.4 mm thickness; (**e**) PAUT inspection result for Line 5: 0.8 mm thickness.

The aforementioned methodology provides a PAUT result as well as a PT result for each delamination line to compare and analyze. Since the PAUT was conducted sequentially, it is preferable to provide the results in a sequential manner as well. Figure 6 illustrates the results of the PAUT based on the thickness of the specimen, ranging from the surface to the deeper layers. The scan strips display a 34 mm width segment of the specimen, which coincides with the region covered by the PAUT probe during acquisition. In general, it is apparent that the PAUT method failed to adequately detect the smallest inclusion (3 mm) for all thicknesses. Conversely, inclusions ranging from 5 to 15 mm are identified as square-shaped formations with varying thicknesses. As the size increases, the form closely resembles a square. However, when the size decreases, the distortion becomes more apparent, resulting in a more circular form.

In particular, Line 3 (Figure 6c) shows limited detection capability for the largest inclusion due to the constraints associated with the near-surface PAUT detection (0.2 mm here). This might be associated with the blind-zone effect, which occurs in ultrasonic inspection caused by aftershocks of the probe for near-surface defects [51].

**Figure 7:**
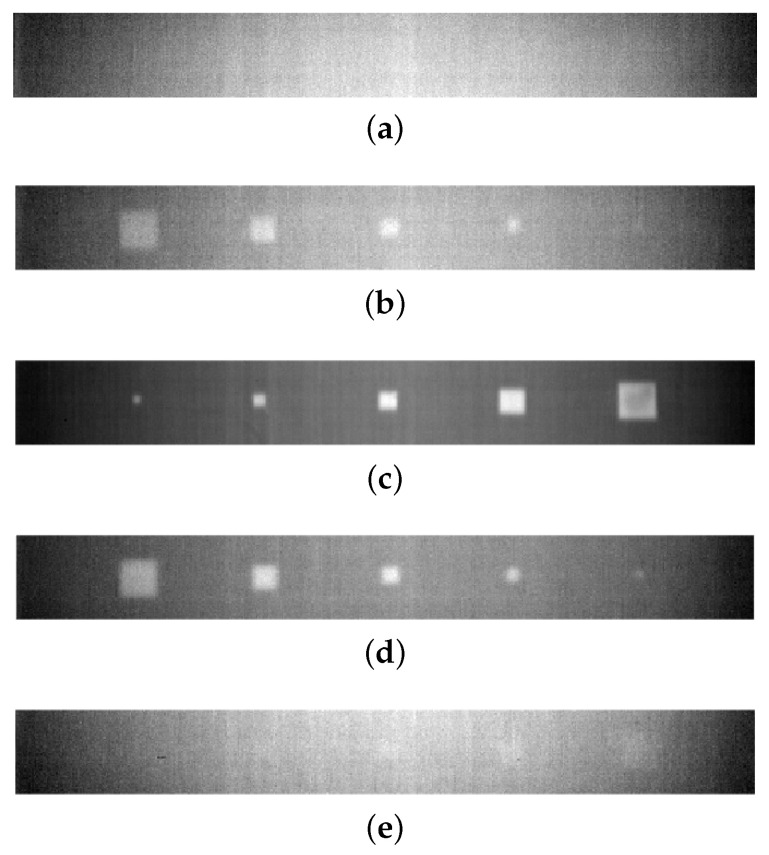
PT inspe ction results for delamination lines with different thicknesses. (**a**) PT inspection result for Line 1: 1 mm thickness; (**b**) PT inspection result for Line 2: 0.6 mm thickness; (**c**) PT inspection result for Line 3: 0.2 mm thickness; (**d**) PT inspection result for Line 4: 0.4 mm thickness; (**e**) PT inspection result for Line 5: 0.8 mm thickness.

Additionally, the pulsed thermography findings for the same lines in five different thicknesses are depicted in Figure 7. In spite of the uneven background due to non-uniform heating, defects are clearly shown in the case of shallow defects (Figure 7c), since the thermal contrast is significantly larger than the non-uniform heating and noise. This situation gradually changes as the depth of defects increases [52]. Specifically, the absence of inclusions is clearly shown in the deepest line within 1 mm (Figure 7a). The relationship between depth and pulse heat penetration is significant, as the pulse heat has not fully permeated the material [53]. The temperature at this particular thickness should be sufficiently constant so as not to create a noticeable difference for inclusion. Similarly, at a depth of 0.8 mm (Figure 7e), there is minimal evidence of detection, save for faint shadows that may be improved through certain processing procedures.

Conversely, it is highly visible that the shallowest line (Figure 7c) exhibits a sharp detection in terms of the sufficient contrast between the sound and defective regions, as well as providing square shapes for all sizes of inclusions. It is important to highlight that the PA inspection findings showed negligible indication of any tiniest inclusions. The remaining two middle lines (Figure 7b,d) likewise exhibit square forms for the observed inclusions. However, the image becomes blurry and deformed when the size of the inclusions decreases.

**Figure 8:**
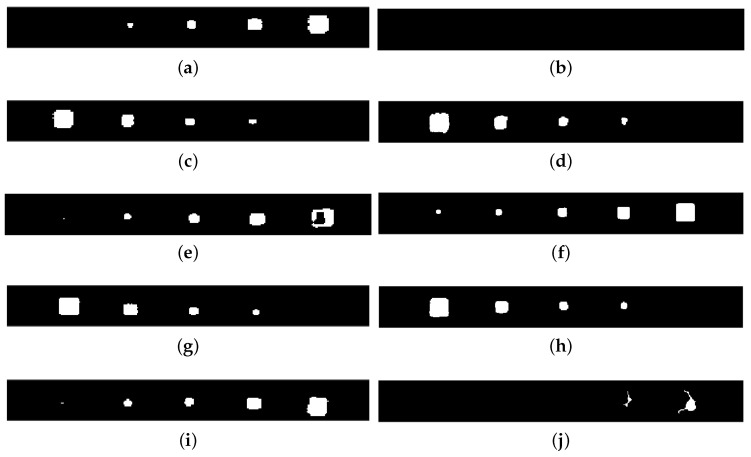
PA and PT inspections’ binary images for delamination lines with different thicknesses. (**a**) PA binary image for Line 1: 1 mm thickness; (**b**) PT binary image for Line 1: 1 mm thickness; (**c**) PA binary image for Line 2: 0.6 mm thickness; (**d**) PT binary image for Line 2: 0.6 mm thickness; (**e**) PA binary image for Line 3: 0.2 mm thickness; (**f**) PT binary image for Line 3: 0.2 mm thickness; (**g**) PA binary image for Line 4: 0.4 mm thickness; (**h**) PT binary image for Line 4: 0.4 mm thickness; (**i**) PA binary image for Line 5: 0.8 mm thickness; (**j**) PT binary image for Line 5: 0.8 mm thickness.

Proceeding with the binary representation of these results of PAUT and PT inspections shown in Figure 8, respectively, they might have a significant impact on the simplicity, efficiency, and distinct differentiation of characteristics, which are key factors to consider. They show an enhanced ability to detect individual inclusions by displaying them with white pixels with the background in black. Here, the first thing to emphasize is that the results from both sources confirm each other in terms of approximate locations, sizes, and shapes, as far as they were able to detect.

In detail, PAUT has a small point detection for the smallest inclusions in Lines 3 and 5. The other four inclusions were detected well for all thicknesses. It must be mentioned here that the rectangular-like shape of the PAUT inspection results is due to the scaling difference from the PT images. This has to be equalled to register and combine information from these modalities. Regarding PT, it is obvious that the two deepest lines lack accurate identification in terms of both size and form. It might be stated that Line 3 is the best visual detection in terms of having all size inclusions and their near-perfect shapes.

In summary, it can be extracted from the results that PT-based near-surface detection provides a more comprehensive knowledge of delamination in our situation, whereas PAUT is definitely more capable of identifying deeper defects, as anticipated.

### 3.3. Fusion

**Figure 9:**
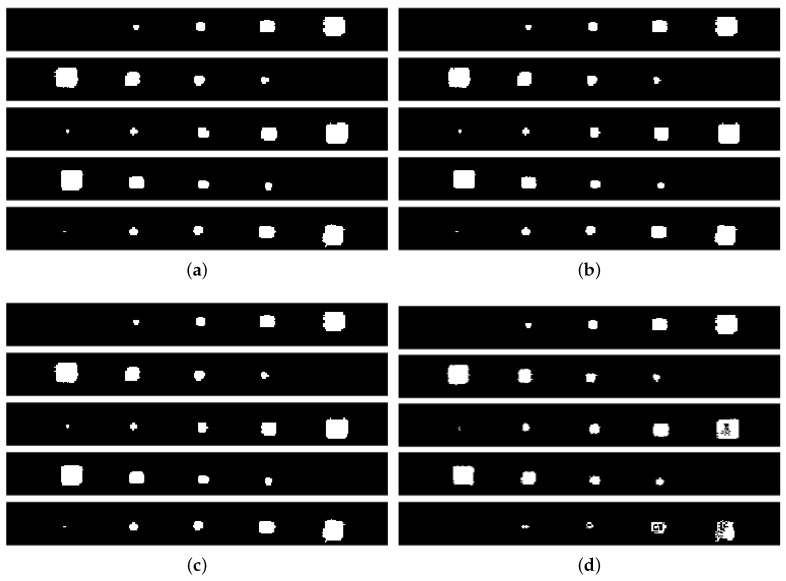
Fusion images with different combination rules for overall detection. (**a**) Maximum fusion rule detection images; (**b**) weighted-average rule detection images; (**c**) DDC rule detection images; (**d**) wavelet decomposition fusion detection images.

In this study, we investigate the procedure and outcomes of applied complementary fusion on two source images that depict a landscape of inspections. The objective is to integrate these inspections in a way that addresses their individual deficiencies and improves the overall portrayal of the detection appearance. The fusion of these two inspection modalities has provided a more holistic detection, taking advantage of both imaging techniques. Figure 9 presents four sets of the fused images regarding their combination rules using the aforementioned individual detection images. In addition, Table 3 gives the mean absolute error (MAE) values between the source images and real inclusions, quantifying the average number of differing pixels. Since the pixel values are unitless, the unitless MAE reflects the average absolute difference in pixels. It is evident that the best near-surface-level detection (Line 3) was provided by PT, whereas only PAUT inspection was capable of detecting deeper layers (Lines 1 and 5). Thus, the individual images exhibit lower MAE ratings compared to the fused images. According to the MAE results, the detection of middle layers (Lines 2 and 4) was enhanced by the use of maximum combination and depth-driven combination rules. The minimum values of MAE are shown in bold.

The process of complementary fusion comprises the blending of information from two distinct sources, capitalizing on their respective strengths. In our scenario, PAUT inspection achieved superior resolution across the entire depth, effectively detecting defects (except for the tiniest ones) in deeper layers of the structure. However, it exhibited shortcomings in detecting anomalies at the level of the surface. Conversely, PT exhibited solid shape and size features for shallower layers, as anticipated, but it did not possess the ability to detect deep layers. Combining individual sources highlights and balances their cooperative detections. Maximum, DDC, weighted-averaging, and wavelet decomposition-based combination rules are applied and given in Figure 9.

Table 4 presents individual detection errors in percentages for the source inspections and fusion images. These errors were calculated based on defect sizes compared to the ground-truth delamination defects. As discussed before, neither modality could provide any information for some of the defects. These values were denoted as ‘ND’—‘no detection’. In addition, the inspection lines were separated into five defects as regions of interest, and then the errors were calculated for them individually. This means that these error values are not generic PA or PT detection errors, but they are specific errors for different sizes of inclusions. Finally, minus (‘−’) values in the table indicate that the detected size is smaller than the real defect size, but positive ones are bigger. The majority of the detected sizes are bigger than the real sizes, according to the table. This shows that the inspection methods tended to overestimate the size of defects. This can be explained by the fact that a defect includes the edge locations where the inclusions come into contact with the main structure. This means that the presence of contact points could potentially introduce overestimation. Identifying the optimal fusion strategy for this particular combination is challenging. It is evident that complementary detection through fusion has provided a better comprehension of overall detection.

Considering that a fusion method is able to combine existing data from individual sources, it was not possible to obtain information for undetected inclusions. As can be seen from the errors of individual sources, the smallest inclusions could mostly not be detected. Although advanced signal processing tools might increase the detectability of these small defects, this study focuses on emphasizing the combined effect rather than using an improvement tool. On the other hand, some of the defects (Line 5 D1 for PA; D4 and D5 for PT) have quite low detection rates, with error rates of over 80 percent. This also shows that these low detections are open to improvement using advanced signal processing tools, which are mentioned in the final section. Typically, it is expected that there will be a higher error in Line 1 (1 mm depth). However, the PT system failed to detect these defects because of the depth. Thus, for Line 5, PT offered insufficient detection markers, resulting in significant error rates.

From another point of view, Defect 3 might be worth investigating; they are all the same size, but have different depths, and the error values seem to be compatible with the detection characteristics and ranges of the source methods. As an exception, for Line 3, which is closest to the surface, while other errors were quite high for PA, Defect 3 showed a value very close to that achieved with PT, interestingly. This can be attributed to the fact that the reflections caused by the flash applied in the PT application are concentrated in the middle section and increase the brightness of the image in the middle area. This higher brightness might be the reason for the higher detection error for that defect in PT specifically. Overall, the applied fusion rules gave the lowest error rates in some regions where the effect of the combination was intense (e.g., DDC in Line 2, D4). Apart from these, the lowest error is seen in PT for the near-surface areas and in PA for deep regions, as expected.

## 4. Conclusions and Future Work

This study investigated the process and outcomes of using complementary fusion on two source images (phased-array ultrasonic and pulsed thermography) that describe the setting of inspections. The goal was to combine these inspections in order to overcome their shortcomings and enhance the overall depiction of detection. The fusion of these two inspection modalities resulted in a more holistic detection, utilizing the strengths of both imaging techniques. The results demonstrate that while individual images may exhibit lower mean absolute error (MAE) ratings naturally for their strongest working area compared to fused images, complementary fusion enhances detection in the overall scenario.

PAUT achieved superior resolution across the entire depth, effectively detecting defects in deeper layers, while PT exhibited solid shape and size features for shallower layers but lacked the ability to detect defects in deep layers. To combine these strengths and balance their cooperative detections, various combination rules were applied. Maximum, depth-driven combination (DDC), weighted-averaging, and wavelet decomposition-based combination rules were explored. It should be pointed out, however, that raw data were exclusively investigated in PT. As is well known, PT results can be significantly improved through the use of advanced signal processing (e.g., pulsed phase thermography (PPT), principal component thermography (PCT), thermographic signal reconstruction (TSR), etc.). For instance, in [54], it was possible to clearly detect 24 of 25 inserts of the same specimen by processing the PT sequences via PPT and retrieving the phase. Hence, it is highly likely that using the proposed PAUT-PT fusion approach but with the phase (instead of with raw thermograms) would produce improved defect detection results after fusion.

Individual detection errors for the source inspections and fusion images were presented and calculated based on defect sizes compared to the ground-truth delamination defects. Some defects (i.e., the smallest-size defects that, in principle, hinder easy detection) remained undetected by either modality. Inspection lines were separated into regions of interest, and errors were calculated for these individually. The majority of detected sizes appeared to be larger than the real size, and this indicates a tendency to overestimate defect sizes in these inspection methods.

Complementary detection through fusion provides improved overall detection that leverages the strengths of each modality for defect detection across the overall thickness of a specimen. Further research might focus on enhancing these fusion techniques, minimizing overestimation, and using advanced signal processing with PT data to improve defect detection capabilities significantly. Moreover, the applied fusion approach can also be extended to similar composite structures, such as GFRPs, if there are no restrictions in terms of detection (such as hollow structures not being suitable for the transmission of ultrasonic signals).

## Figures and Tables

**Table 1:** Specimen and defect descriptions.

Specimen	Defect Material	Defect Sizes	Number of Plies	Thickness (mm)	Size (mm)
**Artificial delamination specimen**	Artificial Teflon inclusions	3, 5, 7, 10, and 15 mm square inclusions (depths and locations indicated in Figure 1)	10	2	300 × 300

**Table 2:** Affine transformation demonstration for scaling, translation, and rotation.

Transformation Type	Identity	Scaling	Translation	Rotation
**Affine Matrix, A**	$\left[\begin{array}{ccc} 1 & 0 & 0\\ 0 & 1 & 0\\ 0 & 0 & 1 \end{array}\right]$	$\left[\begin{array}{ccc} c_{x} & 0 & 0\\ 0 & c_{y} & 0\\ 0 & 0 & 1 \end{array}\right]$	$\left[\begin{array}{ccc} 1 & 0 & t_{x}\\ 0 & 1 & t_{y}\\ 0 & 0 & 1 \end{array}\right]$	$\left[\begin{array}{ccc} cos\theta & -sin\theta & 0\\ sin\theta & cos\theta & 0\\ 0 & 0 & 1 \end{array}\right]$
**Coordinate Equations**	$x^{\prime }=x$ $y^{\prime }=y$	$x^{\prime }=c_{x}x$ $y^{\prime }=c_{y}y$	$x^{\prime }=x+t_{x}$ $y^{\prime }=y+t_{y}$	$x^{\prime }=xcos\theta -ysin\theta$ $y^{\prime }=xsin\theta +ycos\theta$
**Example Shape**	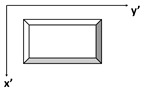	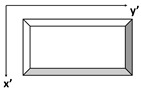	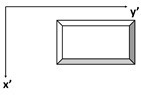	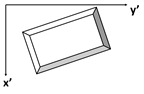

**Table 3:** Mean absolute error (MAE) values between source images and fused images for individual lines.

	Methods for Line Detection	Line1	Line 2	Line 3	Line 4	Line 5
**Sources**	PA	**0.0113**	0.0115	0.0209	0.0112	**0.0131**
	PT	-	0.0109	**0.0062**	0.0301	0.0421
**Fusion Rules**	Maximum	0.0113	**0.0093**	0.0098	**0.0083**	0.0134
	DDC	0.0113	0.0099	0.0090	0.0085	0.0134
	Weighted Averaging	0.0113	0.0109	0.0097	0.0112	0.0131
	Wavelet Decomposition	0.0369	0.0230	0.0310	0.0210	0.0355

Bold values are the minimum values of MAE for related lines

**Table 4:** Defect detection errors for sources and fusion operations based on the defect sizes.

			Detection Errors (%)				
		**Detected by**	**Defect 1**	**Defect 2**	**Defect 3**	**Defect 4**	**Defect 5**
**Line 1**	Sources	PA	ND	−33.33	−11.90	7.65	6.40
		PT	ND	ND	ND	ND	ND
	Fusion Rules	Maximum	ND	−33.33	−11.90	7.65	6.40
		DDC	ND	−33.33	−11.90	7.65	6.40
		Weighted Average	ND	−33.33	−11.90	7.65	6.40
		Wavelet (Daubechies)	ND	−33.33	−11.90	7.65	6.40
**Line 2**	Sources	PA	3.74	0.00	−7.14	−20.00	ND
		PT	13.33	16.47	14.29	−13.33	ND
	Fusion Rules	Maximum	14.94	27.65	19.05	−2.22	ND
		DDC	14.94	27.65	19.05	−2.22	ND
		Weighted Average	3.73	0.00	−7.14	−20.22	ND
		Wavelet (Daubechies)	8.53	8.24	3.57	−17.78	ND
**Line 3**	Sources	PA	−94.00	−13.33	17.86	18.24	−20.27
		PT	13.33	−2.22	21.43	8.24	13.07
	Fusion Rules	Maximum	13.33	26.67	30.95	30.59	21.87
		DDC	13.33	2.22	21.43	20.00	21.87
		Weighted Average	13.33	−2.22	21.43	8.24	13.07
		Wavelet (Daubechies)	−40.00	−6.67	19.05	12.94	−3.74
**Line 4**	Sources	PA	11.47	4.12	−1.19	−11.11	ND
		PT	10.40	1.18	1.19	11.11	ND
	Fusion Rules	Maximum	20.27	17.06	11.90	20.00	ND
		DDC	20.27	17.06	11.90	20.00	ND
		Weighted Average	12.27	2.94	0.00	13.33	ND
		Wavelet (Daubechies)	11.47	4.12	-1.19	0.00	ND
**Line 5**	Sources	PA	−86.67	28.89	−10.71	7.09	−0.27
		PT	ND	ND	ND	−80.00	−66.93
	Fusion Rules	Maximum	-86.67	28.89	−10.71	8.24	1.33
		DDC	−86.67	28.89	−10.71	8.24	1.33
		Weighted Average	−86.67	28.89	−10.71	7.09	−0.27
		Wavelet (Daubechies)	−93.33	−35.56	−55.95	−36.47	−33.60

ND → no detection. Gray color shows the minimum error values.

## Data Availability

The original contributions presented in the study are included in the article, further inquiries can be directed to the corresponding author.

## Abbreviations

The following abbreviations are used in this manuscript:

CFRP	carbon fiber-reinforced polymer
CT	computed tomography
DDC	depth-driven combination
GFRP	glass fiber-reinforced polymer;
MAE	mean absolute error
MMIR	multimodal image registration
NDT&E	non-destructive testing and evaluation
PA	phased array
PAUT	phased-array ultrasonic inspection
PI	pattern intensity
PT	pulsed thermography
SNR	signal-to-noise ratio
SSD	sum of squared differences
